# Patellar shape is associated with femoral trochlear morphology in individuals with mature skeletal development

**DOI:** 10.1186/s12891-022-05000-w

**Published:** 2022-01-17

**Authors:** Lanyu Qiu, Jia Li, Bo Sheng, Haitao Yang, Zhibo Xiao, Fajin Lv, Furong Lv

**Affiliations:** Department of Radiology, The First Affiliated Hospital of Chongqing Medical University, 1 youyi road, yuzhong district, Chongqing, 400016 P.R. China

**Keywords:** Patellar shape, Trochlear morphology, Patellofemoral instability, Knee surgery, Skeletal development

## Abstract

**Background:**

As several studies have detected correlations between patellar and femoral trochlear development, this raises the question of whether patellar shape is associated with trochlear developmental outcomes.

**Methods:**

Patellar shape and femoral trochlear morphology were retrospectively analyzed in 183 subjects, of whom 61 each were classified as having Wiberg type I, II, and III patellae (groups A, B, and C, respectively). The sulcus angle (SA), lateral trochlea inclination angle (LTA), medial trochlear inclination angle (MTA), lateral facet length (LFL), medial facet length (MFL), lateral trochlear height (LTH), medial trochlear height (MTH), trochlea sulcus height (TH), and lateral-medial trochlear facet distance (TD) were analyzed as a means of evaluating trochlear morphology. Trochlear depth, trochlear condyle asymmetry, and trochlear facet asymmetry were additionally calculated, and differences in trochlear morphology and correlations between trochlear morphology and patellar shape were evaluated.

**Results:**

The femoral trochlear parameters of patients in group A differed significantly from those of patients in groups B and C. No significant differences between groups B and C were evident. Patellar shape was positively correlated with LTA, MTA, MFL, trochlear condyle asymmetry, and trochlear facet asymmetry, and was negatively correlated with SA.

**Conclusions:**

These data indicated that patellar shape and trochlear morphology are related to one another,which suggest normalized patella morphology surgery and trochlear surgery are better choices for patients with patella instability.

**Trial registration:**

Retrospectively registered.

## Background

The patellofemoral joint is a key knee component that is comprised of the patella, both active and passive mechanisms maintain patellofemoral joint stability, and many studies have explored the mechanics whereby the patella can track the trochlea during knee movement [1, 2]. Patellofemoral instability occurs with high frequency in both adolescents and adults owing to a range of potential underlying anatomical abnormalities. Primary patella instability is generally treated conservatively, but reoccurrence affects an estimated 17-66% of patients, and is particularly common in those with trochlear dysplasia who exhibit a dislocation rate of up to 69% [3], necessitating surgical intervention. Surgical treatments focus on correcting any underlying anatomical abnormalities and bolstering intrinsic patellofemoral joint stability through approaches such as MPFL reconstruction, tibial tubercle transfer, patellar osteotomy, femoral and tibial osteotomies, and trochleoplasty. However, MPFL reconstruction can result in postoperative complications including recurrent patellar instability [4, 5], primarily because such reconstruction does not correct underlying trochlear dysplasia, and trochlear shape is associated with recurrent patellar dislocation [3]. By restoring the underlying bony anatomy, trochleoplasty can significantly decrease postoperative instability [6]. Even so, the relative benefits of trochleoplasty and the appropriate means of performing this procedure remain controversial. It is thus essential to precisely define the differences in femoral trochlear morphology among patients and to understand how these morphological differences are related to different patellar shapes in order to guide trochleoplasty treatment planning.

Patellar and trochlear development has been widely studied in the context of patellar instability. Two primary theories exist with respect to the development of trochlear morphology. Several studies have found that trochlear sulcus shape is primarily determined during early embryonal stages [7, 8]. However, other studies have suggested that trochlear and patellar development continues even after birth. Yang et al. [9] found that trochlear morphology shifts from normal to dysplastic when insufficient patellar stress was applied to the trochlea in animal model systems, and abnormal positioning of the patella relative to the femoral trochlear sulcus resulted in a more flattened sulcus [10]. Similar findings have also been observed in humans. Richmond et al. [11], for example, found that the patella and trochlea sulcus exhibit concurrent deepening over time prior to 8 years of age. Given that several studies have detected correlations between patellar and femoral trochlear development, this raises the question of whether patellar shape is associated with trochlear developmental outcomes. Bone shape and growth are associated with bone functions [12], and patella tracking is vital to joint development. A change in patellar shape may result in inappropriate trochlear sulcus modeling owing to the interaction between the two.

Trochleoplasty outcomes are associated with trochlear shape. However, correct patellar tracking is essential to facilitate the stability of the patellofemoral joint, suggesting that patellar shape should also be taken into consideration when conducting trochleoplasty. The goal of this study was to analyze femoral trochlear morphological differences associated with different patellar shapes and to study correlations between patellar shape and trochlear morphology. However, no studies to date have been published regarding the association between trochlear and patellar morphology in individuals with mature skeletal development. The aim of this study was therefore to address these limitations by studying a large cohort of patients with different patellar shapes in order to better understand these morphological relationships.

## Methods

### Study Population

**Fig. 1:**
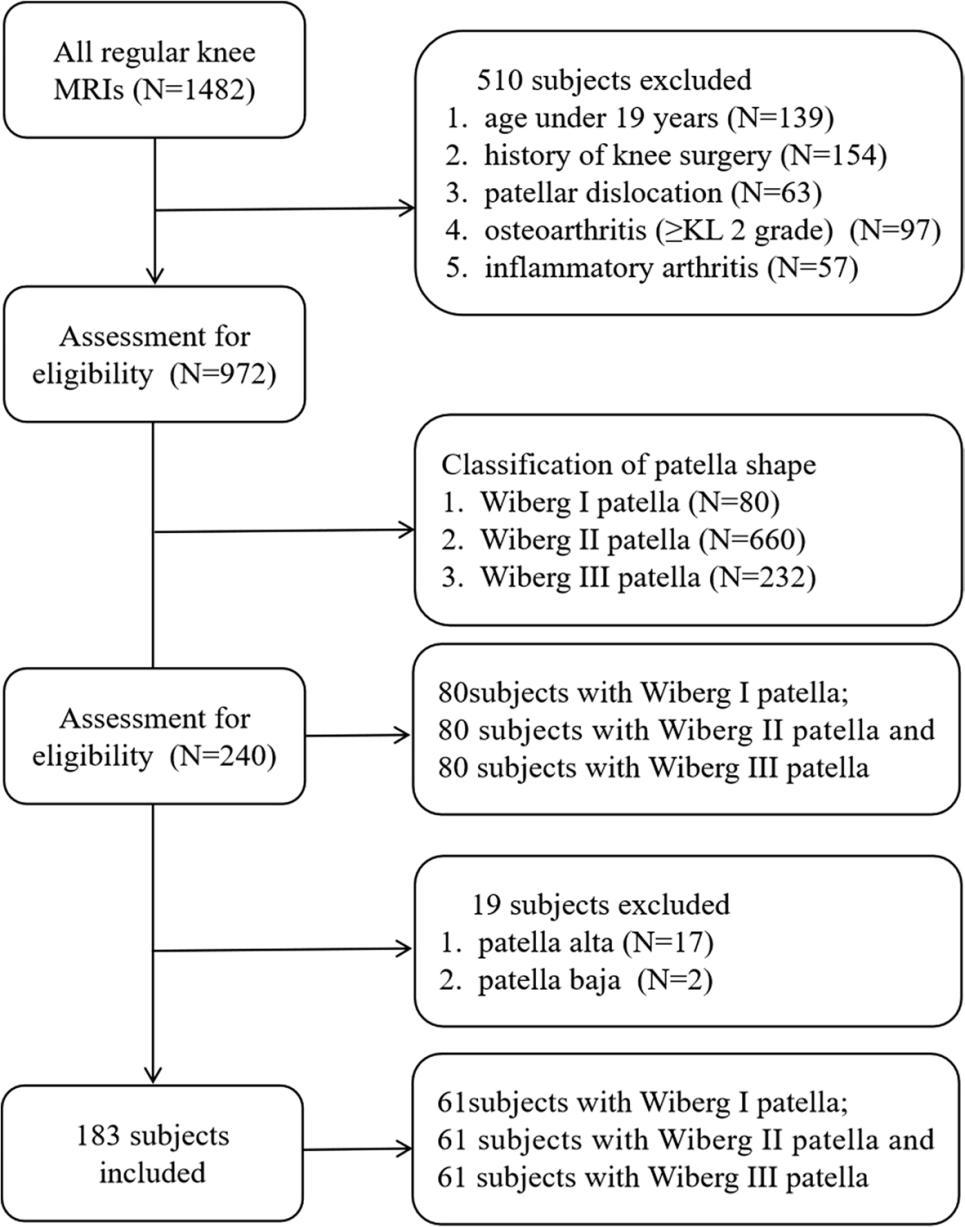
Flowchart for subject selection

The Committee for Human Research of our institution approved the present retrospective study of patients that had undergone magnetic resonance imaging (MRI) scans for either knee pain or other medical examinations between January 2014 and August 2021. Patients were excluded from this study if they (1) were < 19 years old; (2) had a history of knee surgery; (3) suffered from patellar dislocation; (4) exhibited evidence of severe osteoarthritis (≥KL 2 grade) or inflammatory arthritis; or (5) with patella alta or patella baJa. MRI scans and medical records for all subjects were evaluated. The Wiberg classification system was used to divide patients into three groups according to patellar shape, with 80 Wiberg type I patients being incorporated into group A in this study, while 80 age-, gender- and side-matched patients with Wiberg type II and III patellae were each incorporated into groups B and C, respectively. The patient selection process is outlined in Fig. 1.

## MRI Protocol

MRI scans were performed on a 1.5-T MRI equipment (Magnetom Essenza; Siemens Healthcare) with an extremity matrix knee coil (Tim coil) for all subjects. A standard MRI protocol was used, which included sagittal and coronal T1 turbo spin-echo (repetition time [TR], 306 ms; echo time [TE], 12 ms; field of view [FOV], 16 cm; matrix size, 320 × 320; slice thickness, 4 mm), sagittal T2 turbo spin-echo (TR, 3220 ms; TE, 99 ms; FOV, 16 cm; matrix size, 320 × 320; slice thickness, 4 mm), sagittal and coronal intermediate-weighted turbo spin-echo with fat saturation (TR, 2800 ms; TE, 38 ms; FOV, 16 cm; matrix size, 256 × 256; slice thickness, 4 mm), and axial intermediate-weighted turbo spin-echo with fat saturation (TR, 2800 ms; TE, 51 ms; FOV, 16 cm; matrix size, 232 × 256; slice thickness, 4 mm).

## Image Analysis

**Fig. 2:**
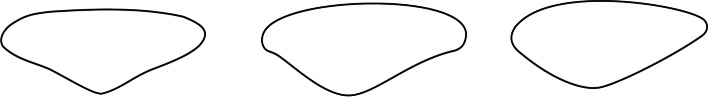
Wiberg classification for patella morphology [13, 14]. Wiberg type I patella (the left):medial facet equal size as lateral facet; Wiberg type II patella (the center) : medial facet smaller than lateral facet; Wiberg type III patella (the right) : medial facet very much smaller than lateral facet

**Fig. 3:**
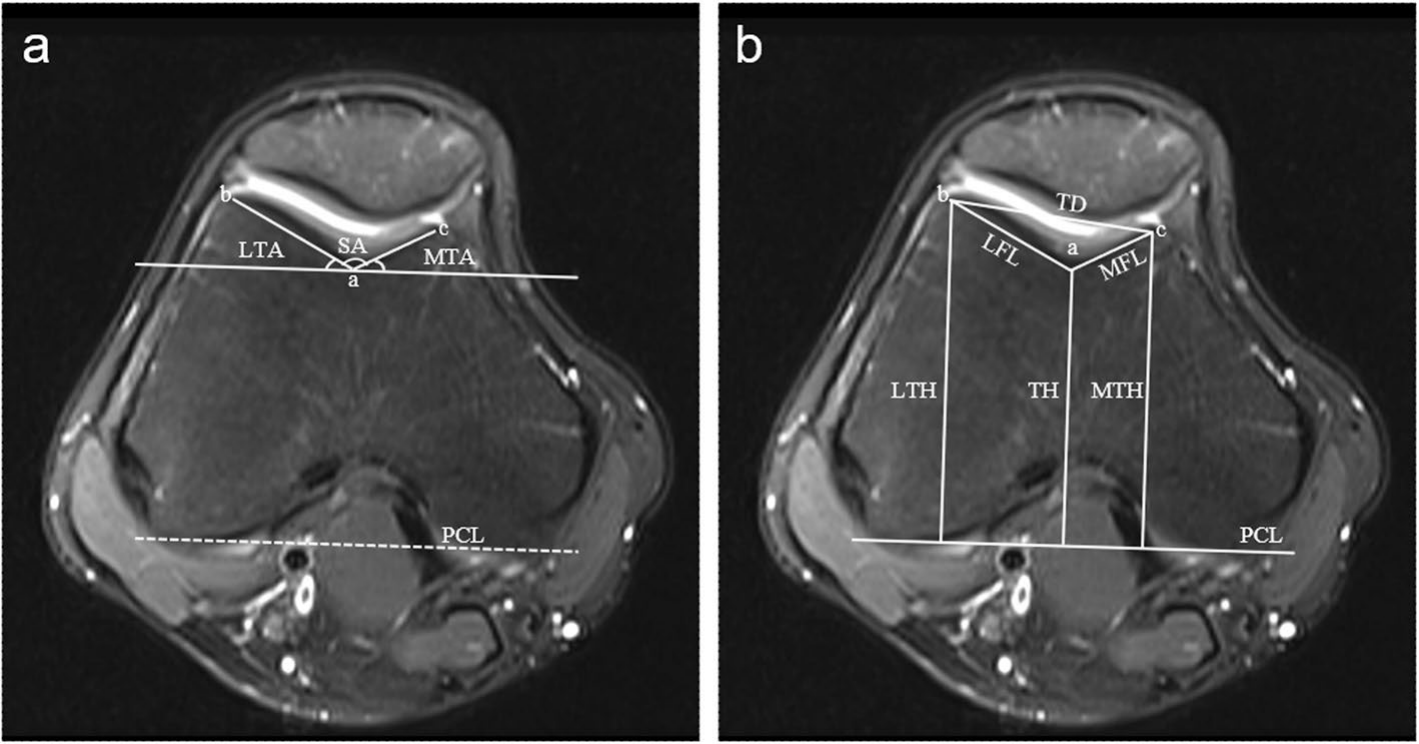
Measurements on the transverse image obtained 3 cm above the femorotibial joint space. (**A**) The angle of trochlear facet. The SA was the angle between the ab and ac; LTA and MTA were the angle between the posterior condyle line (PCL) and the ab and ac respectively. a: the deepest point of the trochlear sulcus; b and c: the point of the most prominent point of the lateral and medial femoral condyle. (**B**) The parameters of trochlear morphology. LFL was the distance between a and b. MFL was the distance between **a** and **c**. TD was the distance between **b** and **c**. TH, LTH and MTH were the vertical distance from a, b and c to PCL. Note: LFL lateral facet length, LTA lateral trochlea inclination angle, LTH lateral trochlear height, MFL medial facet length, MPFL medial patellofemoral ligament, MTA medial trochlear inclination angle, MTH medial trochlear height. PCL posterior condyle line, TD lateral-medial trochlear facet distance, TH trochlea sulcus height, SA sulcus angle

Two radiologists with 12 and 18 years of respective clinical experience who were blinded to this study conducted all image analyses. The Wiberg system [13, 14] was used to classify patellar shape (Fig. 2). Femoral trochlea parameters were measured on the transverse image obtained 3 cm above the femorotibial joint space where development of the femoral trochlea could be reliably evaluated [15]. Measured parameters included: the sulcus angle (SA), lateral trochlea inclination angle (LTA), medial trochlear inclination angle (MTA), lateral facet length (LFL), medial facet length (MFL), lateral trochlear height (LTH), medial trochlear height (MTH), trochlea sulcus height (TH), and lateral-medial trochlear facet distance (TD). Subsequent measurements were defined using the posterior condyle line (PCL) as a baseline. All measurements were based on bone, given that cartilaginous trochlear morphology differs from that of the underlying bony trochlea in those affected by trochlear dysplasia. The SA was assessed by measuring the angle between the line of lateral and medial facet inclination. The LTA and MTA were measured as angles between the facet inclination and the PCL. The LFL and MFL were measured based upon the distance between the most prominent point of the lateral and medial femoral condyle and the deepest point of the trochlear sulcus. The LTH and MTH were defined as the maximum anteroposterior distance of the lateral and medial femoral condyle and the PCL. The TH was defined as the minimum anteroposterior distance from the deepest point of the trochlear sulcus to the PCL. Trochlear depth was calculated as follows: depth=(MTH+LTH)/2-TH. Trochlear condyle asymmetry and trochlear facet asymmetry were respectively calculated according to the following formula [16] :TCA=MTH/LTH×100% and TFA=MFL/LFL×100% (Fig. 3).

### Statistical Analysis

SPSSwas used for statistical testing. Categorical variables including sex and side were assessed using chi-squared tests. Continuous variables such as age and trochlear parameters were described using appropriate descriptive statistics, and, when normally distributed, were expressed as means ± standard deviation and compared via one-way ANOVAs and least-significant difference tests. Relationships between patellar shape and trochlear morphology were assessed using Spearman’s rank correlations. P < 0.05 was the significance threshold for this study. Intra- and interobserver reliability were assessed based on an intraclass correlation coefficient (ICCs) and 95% confidence interval, with values of 0.8 – 0.9 and > 0.9 being considered good and excellent, respectively.

## Results

In total, 183 patients met with study inclusion and exclusion criteria. The ages of the three groups were 37.1± 9.1 years in group A, 36.5 ± 9.5 years in group B, and 36.6 ± 9.2 years in group C. There were 3 left knee joints and 58right knee joints in group A, 4 left knee joints and 57 right knee joints in group B, and 8 left knee joints and 53 right knee joints in group C. There were 28 women and 33 men in group A, 31 women and 30 men in group B, and 24 women and 37 men in group C. There were no significant differences in age, sex, or site between groups (Table 1). The intra-observer ICCs for the quantitative measurements ranged from 0.865 to 0.976, whereas the inter-observer ICCs ranged from 0.869 to 0.983, consistent with good intra- and inter-reader reliability (Table 2). Significant differences in trochlear parameters were observed between group A and groups B and C (Table 3). The SA of patients in group A was (138.7 ± 9.4)° and was smaller than that of patients in group B (147.0 ± 7.6)° and group C (146.9 ± 8.6)° (*P* < 0.001). The inclination angles of the lateral and medial sides in group A were (20.5 ± 4.7)° and (20.9 ± 7.3)°, respectively, with these values being greater than those in groups B and C. The MFL in group A was (17.1 ± 2.7) mm wider than that in groups B and C (*P* < 0.001). Additionally, greater trochlear facet asymmetry, trochlear condyle asymmetry, and trochlear depth were observed in group A(*P* < 0.001) (Table 4). No trochlear parameters differed significantly between groups B and C.

**Table 1:** Demographic factors of the patients

Characteristic	Group A	Group B	Group C	t value/ χ2 value	P value
Age, yr	37.1± 9.1	36.5 ± 9.5	36.6 ± 9.2	0.075	0.92
Gender					
Male	33	30	37	1.632	0.442
Female	28	31	24		
Side					
Left	3	4	8	3.05	0.218
Right	58	57	53		

**Table 2:** Inter- and intra-observer ICCs of parameters of trochlea morphology

Measurement	Inter-observer ICCs	Intra-observer ICCs
SA	0.942 (0.878-0.972)	0.904 (0.799-0.955)
LTA	0.902 (0.795-0.953)	0.886 (0.761-0.946)
MTA	0.869 (0.724-0.937)	0.865 (0.716-0.936)
LFL	0.930 (0.854-0.967)	0.951 (0.898-0.977)
MFL	0.958 (0.912-0.980)	0.942 (0.877-0.972)
LTH	0.983 (0.965-0.992)	0.975 (0.947-0.988)
TH	0.983 (0.965-0.992)	0.974 (0.949-0.988)
MTH	0.977 (0.951-0.989)	0.976 (0.949-0.988)
TD	0.961 (0.918-0.982)	0.975 (0.948-0.988)

**Table 3:** Results of morphometric parameters in the groups

Measurement	Group A	Group B	Group C	Statistical analysis	
				F	P
SA (°)	138.7 ± 9.4	147.0 ± 7.6	146.9 ± 8.6	18.937	0.000
LTA (°)	20.5 ± 4.7	16.9 ± 4.4	17.4 ± 4.7	11.300	0.000
MTA (°)	20.9 ± 7.3	15.6 ± 6.5	15.8 ± 7.5	10.733	0.000
LFL (mm)	22.3 ± 2.2	22.6 ± 2.3	22.5 ± 2.6	0.1564	0.858
MFL(mm)	17.1 ± 2.7	15.0 ± 2.7	14.5 ± 2.5	16.540	0.000
LTH (mm)	57.8 ± 4.9	57.8 ± 5.2	57.7± 4.9	0.003	0.997
MTH (mm)	56.0 ± 5.1	54.7 ± 4.8	54.5 ± 4.4	1.629	0.199
TH (mm)	50.0 ± 4.5	50.8 ± 4.7	50.9 ± 4.3	0.758	0.470
TD (mm)	36.4 ± 3.6	35.5 ± 3.2	35.1 ± 3.4	2.469	0.088

**Table 4:** Results of trochlear parameters in the groups

Measurement		Group A	Group B	Group C	Statistical analysis	
					F	P
trochlear depth (mm)		6.9 ± 1.8	5.4 ± 1.3	5.3 ± 1.7	14.076	0.000
trochlear condyle asymmetry (%)		96.9 ± 4.1	9.48 ± 4.2	94.7 ± 4.4	5.539	0.005
trochlear facet asymmetry (%)		76.7 ± 12.4	67.0 ± 13.2	65.0 ± 13.3	19.060	0.000

Patellar shape was significantly correlated with trochlear parameters including SA, LTA, MTA, MFL, trochlear depth, trochlear facet asymmetry, and trochlear condyle asymmetry.The symmetry of the patella medial and lateral articular surfaces differed between groups A, B, and C. Group A has the best symmetry, group B was the second, and group C exhibited the worst symmetry. Patellar facet symmetry was correlated with the aforementioned parameters and was not consistently associated with others. SA was inversely correlated with the patellar facet symmetry (*r* = -0.366 *P* <0.001). Additionally, patellar facet symmetry was positively correlated with LTA (*r* = 0.260 *P* <0.001), MTA (*r* = 0.284, *P* <0.001), MFL (*r* = 0.360, *P* <0.001), trochlear depth (*r* = 0.354, *P* <0.001), trochlear facet asymmetry (*r* = 0.362, *P* <0.001) and trochlear condyle asymmetry (*r* = 0.213, *P* =0.004).

## Discussion

The primary finding of the present study was that there was a significant correlation between patellar shape and trochlear morphological parameters. The LTA, MTA, MFL, trochlear depth trochlear condyle asymmetry, and trochlear facet asymmetry were increased in subjects with Wiberg type I patellae relative to those with Wiberg type II and III patellae, whereas the opposite finding was observed for the SA in these patients. No significant differences in trochlear morphology were observed when comparing those with type II and III patellae. Correlation analyses revealed that individuals with a more symmetric patellar facet exhibited increased trochlear facet asymmetry and trochlear condyle asymmetry. This suggests an interaction between the patella and trochlear morphology during skeletal development, providing valuable information that can aid in selecting appropriate surgical procedures for treating patellofemoral joint instability.

The Wiberg classification system defines three primary patellar shapes based upon the size relationship between the medial and lateral patellar facets [13]. We observed different trochlear morphological parameters in those with Wiberg type I patellae relative to those with Wiberg type II and III patellae. Those with type I patellae exhibited equal medial and lateral facet lengths and the most symmetric facets. The MFL in group A was increased whereas the LFL was similar relative to other groups such that trochlear facet asymmetry (defined as MFL/LFL) was significantly increased. This suggests that trochlear and patellar morphology changed in a consistent manner across patients. In a study conducted by Fucentese et al. [14], patients with a dysplastic trochlea exhibited a smaller patellar size and an asymmetrical patellar facet. The SA was also evaluated in the present study, revealing that femoral trochlear dysplasia was reproducible and decreased in group A patients, explaining why type I patellae are more stable and associated with a lower incidence of patellar dislocation relative to type II and III patellae.

We detected correlations between patellar shape and femoral trochlear morphology, suggesting that the morphological parameters of these structures interact during development. Several studies have explored patellar and trochlear development, yielding inconsistnet findings. Some reports have suggested that congenital factors regulate femoral trochlear shape [7], whereas other studies have shown the trochlea and patella to develop continuously over time [10, 14, 17, 18]. Fucentese et al. reported morphologic changes in the patellae of trochlear dysplasia patients, detecting molding activity between patellar and trochlear development [14]. In a study of skeletally immature cadavers, concurrent changes in patellar and trochlear shape were detected, consistent with our results and suggesting that these two structures may influence one another during development. We found patellar facet symmetry to be related to trochlear inclination and trochlear facet and condyle asymmetry, indicating that the medial patella is the primary portion that interacts with the trochlear. The mature patellae exhibited lateral facet predominance [19], whereas the medial and lateral patellar facets are equal in size during embryonic development, with medial trochlear morphology variability increasing with age [11]. Biedert et al. also found trochlear dysplasia was mainly located in the center and/or medial trochlea rather than the lateral trochlea [20].

In this study, we found that type I patellae, which exhibited nearly equal medial and lateral facet lengths, had more symmetrical trochlear parameters than type II or III patellae. Patients with increasing medial patellae facets exhibit a concurrent increase in trochlear morphological parameters, suggesting a relationship between patellofemoral structural and functional factors during development [17, 21]. Correct patellar tracking alters patellofemoral joint function and plays a key role in regulating knee joint stability. A deep sulcus and a high lateral trochlea can help to maintain normal patella tracking [6], while abnormal tracking can mold patellar and trochlear morphology and impact patellofemoral joint instability [22]. In the study of Otto et al. [23], patients sufering from patellofemoral instability had a relatively larger lateral patellar facet, which was consistent with this study. Causes of trochlear dysplasia may include developmental factors or false patellar tracking during childhood [10, 22]. Stabilization produces changes throughout the knee range of movement, including both bony and soft tissue constraints. Bony constraint is particularly important after the first 20° of knee flexion [2], while the patella begins to engage with the trochlea as the knee flexes to 30°. Poor patellar and trochlear tracking can give rise to trochlear and/or patellar dysplasia [11, 22]. In this report, we observed concurrent patellar and trochlear changes, possibly due to such patellar tracking during skeletal development.

Trochlear and patellar morphology serve to constrain patellofemoral joint instability, and can contribute to patellar dislocation. While trochleoplasty is an effective means of remodeling the trochlea to address patellar instability, it is rarely performed in clinical settings as it is a complex procedure and little information regarding its efficacy as a function of patellar and trochlear morphology is currently available. With sufficient morphological information, trochleoplasty may become a more viable means of addressing joint instability. In the present study, we found that the patellar and trochlear facets were matched in individuals with mature skeletal development such that a more symmetrical patella facet corresponded to a more symmetrical trochlear facet. It is important to take this into account when conducting trochleoplasty, as performing this procedure based on the shape of the patella and the trochlea rather than solely on the shape of the trochlea may reduce the risk of postoperative complications. Based on the above, normalized patella morphology surgery and trochlear surgery are better choices for patients with patella instability.

There are multiple limitations to this study. For one, we did not evaluate the position of the patella relative to the trochlea, and such positioning may impact the shape of these structures. Second, we did not measure patellar morphological parameters as this study was primarily focused on the relationship between patellar shape and trochlear morphology. Third, all measurements made herein were based on bony structures, and cartilaginous structures of the patellofemoral joint were not included. Finally, we only evaluated patients with mature skeletal development and thus cannot draw conclusions regarding whether the relationships between patellar shape and trochlear morphology arose during embryonic development or after birth. Future research will thus be needed to expand on this study and to address these points.

## Conclusions

In summary, our data revealed a direct relationship between patellar shape and trochlear morphology. Developing a more comprehensive understanding of this relationship will enable clinicians to select suitable surgical procedures for the treatment of patients suffering from patellofemoral joint instability.

## Data Availability

The datasets used and/or analysed during the current study are available from the corresponding author on reasonable request.

## Abbreviations

LFL: Lateral facet length
LTA: Lateral trochlea inclination angle
LTH: Lateral trochlear height,
MFL: Medial facet length
MPFL: Medial patellofemoral ligament
MTA: Medial trochlear inclination angle
MTH: Medial trochlear height
PCL: Posterior condyle line
TD: Lateral-medial trochlear facet distance
TH: Trochlea sulcus height
SA: Sulcus angle
